# Changes in Colonic Bile Acid Composition following Fecal Microbiota Transplantation Are Sufficient to Control *Clostridium difficile* Germination and Growth

**DOI:** 10.1371/journal.pone.0147210

**Published:** 2016-01-20

**Authors:** Alexa R. Weingarden, Peter I. Dosa, Erin DeWinter, Clifford J. Steer, Megan K. Shaughnessy, James R. Johnson, Alexander Khoruts, Michael J. Sadowsky

**Affiliations:** 1 Department of Microbiology and Immunology, University of Minnesota, St. Paul, Minnesota, United States of America; 2 The BioTechnology Institute, University of Minnesota, St. Paul, Minnesota, United States of America; 3 Department of Medicinal Chemistry—Institute for Therapeutics Discovery and Development, University of Minnesota, Minneapolis, Minnesota, United States of America; 4 Department of Medicine, University of Minnesota, Minneapolis, Minnesota, United States of America; 5 Department of Genetics, Cell Biology, and Development, University of Minnesota, Minneapolis, Minnesota, United States of America; 6 Department of Medicine, Division of Infectious Diseases, University of Minnesota, Minneapolis, Minnesota, United States of America; 7 Minneapolis Veterans Affairs (VA) Healthcare System, Division of Infectious Diseases, Minneapolis, Minnesota, United States of America; 8 Department of Medicine, Division of Gastroenterology, University of Minnesota, Minneapolis, Minnesota, United States of America; 9 Center for Immunology, University of Minnesota, Minneapolis, Minnesota, United States of America; 10 Department of Soil, Water & Climate, University of Minnesota, St. Paul, Minnesota, United States of America; University of Arizona, UNITED STATES

## Abstract

Fecal microbiota transplantation (FMT) is a highly effective therapy for recurrent *Clostridium difficile* infection (R-CDI), but its mechanisms remain poorly understood. Emerging evidence suggests that gut bile acids have significant influence on the physiology of *C*. *difficile*, and therefore on patient susceptibility to recurrent infection. We analyzed spore germination of 10 clinical *C*. *difficile* isolates exposed to combinations of bile acids present in patient feces before and after FMT. Bile acids at concentrations found in patients’ feces prior to FMT induced germination of *C*. *difficile*, although with variable potency across different strains. However, bile acids at concentrations found in patients after FMT did not induce germination and inhibited vegetative growth of all *C*. *difficile* strains. Sequencing of the newly identified germinant receptor in *C*. *difficile*, CspC, revealed a possible correspondence of variation in germination responses across isolates with mutations in this receptor. This may be related to interstrain variability in spore germination and vegetative growth in response to bile acids seen in this and other studies. These results support the idea that intra-colonic bile acids play a key mechanistic role in the success of FMT, and suggests that novel therapeutic alternatives for treatment of R-CDI may be developed by targeted manipulation of bile acid composition in the colon.

## Introduction

*Clostridium difficile* infection (CDI) has become one of the most common nosocomial infections in developed countries over the last 20 years, and more recently has become an important cause of community-acquired infectious colitis [1–7]. Antibiotic therapy is the primary trigger for CDI, and it commonly perpetuates its recurrence due to continued and progressive disruption of normal gut microbiota function [1, 8–10].

Fecal microbiota transplantation (FMT), in which fecal microbial communities from a healthy donor are delivered into the GI tract of a patient, has emerged as a highly effective treatment to break the cycle of CDI recurrence [11–14]. However, the mechanisms by which it cures recurrent CDI (R-CDI) remain poorly understood. It is known that the fecal microbiota of R-CDI patients undergoes compositional normalization following FMT [13, 15–21], which is associated with functional restoration of secondary bile acid metabolism mediated by colon bacteria [22]. Some primary bile acids, such as taurocholate (TA), are potent germinants for *C*. *difficile* spores, while certain secondary bile acids act as inhibitors of both germination and vegetative growth of the bacterium [23–27]. However, a model where primary acids promote CDI, while secondary bile acids inhibit CDI following FMT, may be too simplistic. Indeed, chenodeoxycholate, one of the major primary bile acids, is an inhibitor of *C*. *difficile* spore germination [24]. In contrast, deoxycholate, one of the major secondary bile acids, can stimulate *C*. *difficile spore* germination [23, 28]. Furthermore, various *C*. *difficile* isolates have different responses to bile acid induced spore germination [27].

In this report, we tested the effects of combinations of bile acids representative of those found in the feces of R-CDI patients prior to FMT on spore germination and vegetative growth of *C*. *difficile*. In addition, we examined whether the variations in germination response across different strains of *C*. *difficile* might be related to variation in the germinant receptor, CspC. Our results support the hypothesis that changes in colonic bile acid composition associated with FMT can inhibit CDI recurrence. The implication supports development of novel pharmacologic interventions or defined microbial consortia as therapeutics for R-CDI.

## Materials and Methods

### Isolation and characterization of *C*. *difficile* isolates

Isolation of *C*. *difficile* from environmental samples was done using a protocol developed by the CDC and modified for the present study. Sterile phosphate buffered saline, pH 7, with 0.1% Tween 80 (50 mL) was added to sterile bags containing environmental sample sponges. Bags were placed into a Stomacher 400 circulator (Seward Laboratory Systems, Davie, FL) and macerated at 260 RPM for 1 min. The liquid was removed and centrifuged at 3500x*g* for 15 min. The pellet was resuspended in the remaining buffer and a 0.2 mL aliquot of the resulting suspension was plated, in duplicate, onto pre-reduced cycloserine-cefoxitin-fructose agar with horse blood and taurocholate (CCFA-HT, Anaerobic systems, USA). A 1 mL aliquot of the suspension was also inoculated into cycloserine-cefoxitin-fructose broth (CCFB) [29] and CCFA-HT plates and CCFB tubes were incubated for 48–72 h at 37°C, under anaerobic conditions. The *C*. *difficile* colonies from CCFA-HT plates were identified using McLung Toabe agar (lecithinase and lipase-negative), blood agar (no hemolysis), PRO kit (Remel, USA), and Gram staining (Gram-positive spore forming bacilli). Presumptive *C*. *difficile* colonies were further characterized by PCR detection of the pathogenicity locus (PaLoc), binary toxin (*cdtB*), and *C*. *difficile* toxin regulator *tcdC* genes; toxinotyping; and sequence analysis of the *tcdC* gene for specific base pair deletions [30–32]. Confirmed *C*. *difficile* isolates also underwent pulsed-field gel electrophoresis (PFGE) analysis, allowing assignment to North American pulsotypes (NAP) based on an 80% similarity threshold in comparison with CDC reference profiles [33]. Confirmed isolates were stored in 25% glycerol at -80°C until used.

### *C*. *difficile* spore isolation

*C*. *difficile* cells from frozen stocks were inoculated into CCFB medium and grown anaerobically at 37°C for 48 h. Cultures were plated onto brain heart infusion, supplemented with 5 g/L yeast extract and 0.1% L-cysteine (BHIS), and grown for 7 d at 37°C under anaerobic conditions. Colonies from each plate were scraped into 1 mL of ice-cold water and incubated at 4°C overnight to release spores [25]. A 3 mL aliquot of this suspension was loaded onto 10 mL of 50% (w/v) sucrose, in a 15 mL conical tube, and centrifuged in a swinging bucket rotor at 3200 x *g* for 20 min at 4°C. Sucrose and vegetative cells above the spore pellet were removed, and the pellet was washed 5-times in ice-cold H_2_O to remove remaining sucrose. Spores were examined under phase-contrast microscopy to determine purity. Spore samples with > 99% purity (<1% vegetative cells) were stored at 4°C and used in our studies.

### *C*. *difficile* spore germination assays

Germination assays were done as previously described by Sorg and Sonenshein [25]. Spores were heated to 65°C for 30 min and inoculated into BHIS, with or without bile acids, within an anaerobic bag flushed and filled with N_2_ gas. The OD_600_ was measured initially (OD6_00_(t_0_)) and every minute for 20 min (OD_600_(t)) using an EL808 Microplate Reader (Biotek Instruments, Inc., Winooski, VT). Relative OD_600_ for each time point was calculated as OD_600_(t)/OD_600_(t_0_). Experiments were performed in triplicate.

### Growth of *C*. *difficile* vegetative cells

Cells from frozen stocks were inoculated into BHIS broth with 0.1% (w/v) taurocholate and incubated overnight at 37°C under anaerobic conditions. Vegetative cells were inoculated into tubes containing BHIS, with or without bile acids, normalized to OD_600_ = 0.005, and grown anaerobically at 37°C. Measurements of OD_600_ were collected each hour for 12 h following inoculation, and the final OD_600_ was measured after 24 h growth. Experiments were performed in triplicate.

### Sequencing of *C*. *difficile* germinant receptor

Two sets of polymerase chain reaction (PCR) primers were developed to amplify CspC and were designed based on a published DNA sequence of the *C*. *difficile* germinant receptor (Francis et al., 2013) (S1 Table). For each primer set, three PCR reactions were run in order to generate ~700 bp overlapping fragments, which were subsequently used for Sanger sequencing. Samples were amplified in 50 μL reactions containing 1x PCR buffer with 1.5 mM magnesium chloride, 0.2 μM nucleotides, 0.4 μM one forward and reverse primer, 2.5 U Choice Taq (Denville Scientific, South Plainfield, NJ), and 25 ng of template. Reactions were heated to 95°C for 3 min, then subjected to 25 cycles of 95°C for 30 sec, 51°C (forward primers 1A, 1B, and 1C), 57°C (primer 2B), or 58°C (primers 2A and 2C) for 30 sec, and 72°C for 150 (primers 1A and 2A), 90 (primers 1B and 2B), or 60 (primers 1C and 2C) sec, before a final elongation step at 72°C for 15 min. Amplicons were purified with the QIAquick PCR purification kit (Qiagen, Germantown, MD) and sequenced at the University of Minnesota Genome Center. Sequences were manually edited using Chromas Lite (Technelysium Pty Ltd, Australia) and assembled into full-length *cspC* sequences, and translated into protein sequences using the ExPASy protein translate tool (Gasteiger et al., 2003). Protein sequences were aligned and UPGMA trees were generated (with boot-strapping, 1000 iterations) using Clustal X [34].

### Reagents

Sodium taurocholate, sodium cholate, sodium deoxycholate, chenodeoxycholic acid (CDCA), and lithocholic acid (LCA) were obtained from Fisher Scientific (Waltham, MA). The CDCA and LCA were dissolved in 100% ethanol prior to use.

### Statistical analysis

Analysis of variance (ANOVA), followed by Tukey’s honest significant difference (HSD) test, was used to determine statistical significance of germination and growth assay results. All analyses were done at α = 0.05.

### Study approval

The study was approved by the University of Minnesota Institutional Review Board (IRB). Collection of household *C*. *difficile* isolates was performed under IRB 1112M07323. The study of fecal specimens was performed under IRB 0901M56962. All human subjects provided written informed consent prior to participation in the study.

## Results

### Bile acids at concentrations found in feces before FMT induce germination of *C*. *difficile* spores

An established spectrophotometric assay that relies on the refractive nature of bacterial spore coats and cortex was used to examine germination of *C*. *difficile* [23–27]. Spore coats and cortex are refractive towards light and appear bright under phase-contrast microscopy, but are rapidly degraded during the initial step of germination and become phase-dark with a concomitant decrease in light absorption at 600nm (OD600) [35].). In a test of this system using one strain, a 20 min exposure to 2 mM TA (a germinant) in BHIS corresponded to an increase of 69.1% phase-dark and vegetative cells, compared to spores exposed to BHIS alone. In addition, 20 min exposure to 2 mM TA resulted in a 0.1% increase in colony forming units, whereas there was no detectable growth of cells exposed to BHIS alone. We used this germination assay to determine whether bile acids at the same concentrations as that found in patient feces, before and after FMT (Table 1), induced germination of *C*. *difficile* spores.

**Table 1:** Average concentration of bile acids measured in patient feces before and after FMT and concentration used in bile acid combinations.

Bile acid	Concentration (mM)^a^	
	Pre-FMT	Post-FMT
Taurocholate (1^0^)^b^	0.55±0.25	-^c^
Cholate (1^0^)	1.45±0.29	-
Chenodeoxycholic acid (1^0^)	0.37±0.09	-
Deoxycholate (2^0^)^b^	*-*	1.24±0.24
Lithocholic acid (2^0^)	-	0.95±0.15

^a^Mean fecal concentration, n = 12 patients [22] and standard concentration used in this work.

^b^1^0^ = primary bile acid, 2^0^ = secondary bile acid.

^c^Bile acid undetected or < 1% of total bile acids; not used in experimental combination.

**Fig 1:**
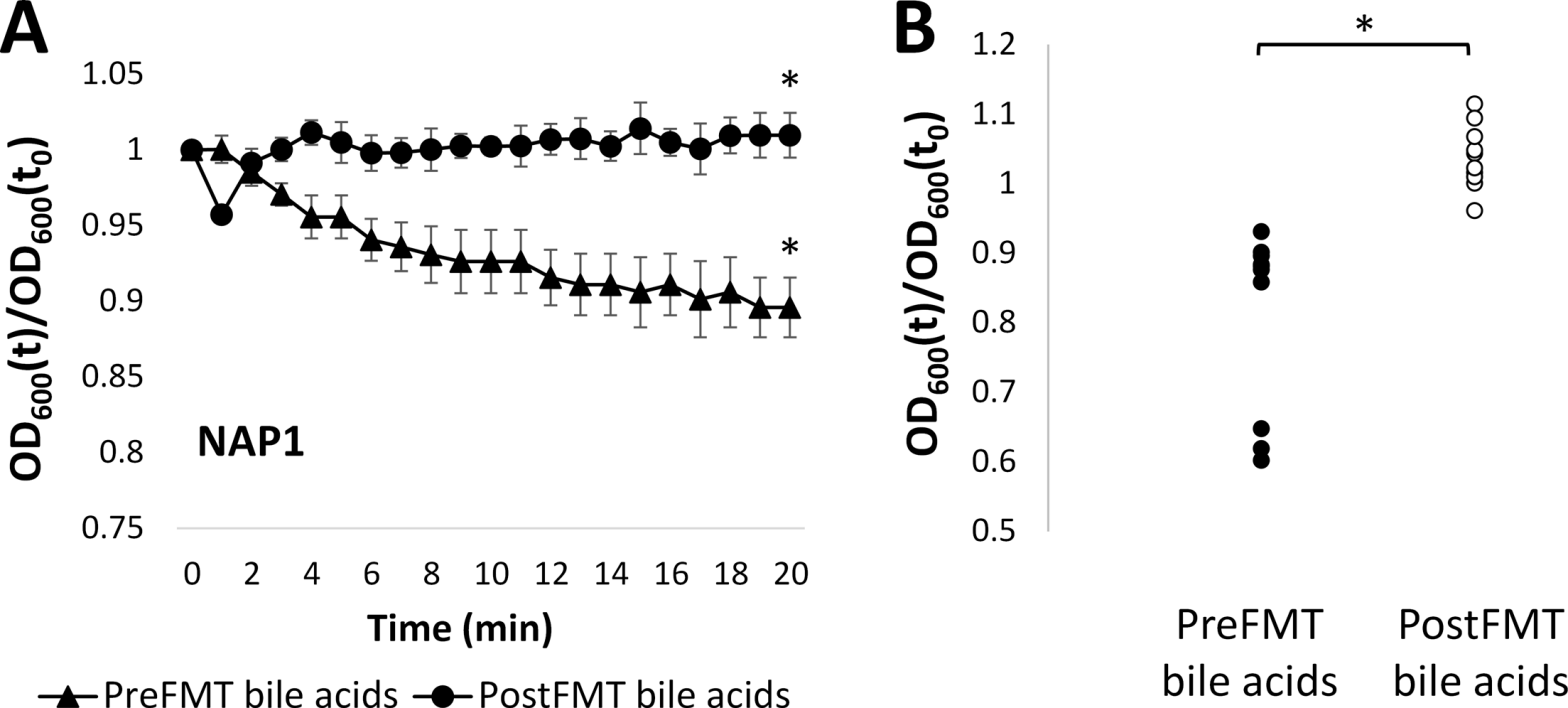
*C*. *difficile* spores germinate in response to pre-FMT fecal bile acids. A) Relative OD_600_ of NAP1 spores following exposure to bile acids present before (triangle) or after (circle) FMT. B) Relative OD_600_ of spores from 10 isolates after 20 min of exposure to bile acids present before or after FMT. OD_600_(t)/OD_600_(t_0_) = OD_600_ normalized to initial OD_600_ (relative OD_600_). * = p < 0.05. Data represent mean ± standard error on mean (SEM).

We previously demonstrated that treatment of R-CDI patients with FMT results in conversion of fecal bile acid composition from domination by primary bile acids to one dominated by secondary bile acids [22]. Here we exposed *C*. *difficile* spores from a North American Pulsed-field gel electrophoresis type 1 (NAP1) strain, which is considered hypervirulent [36], to combinations of bile acids at concentrations measured in patient feces before and after FMT (pre- and post-FMT bile acids, respectively) (Table 1). Results in Fig 1A show that the relative OD_600_ of spores exposed to pre-FMT bile acids significantly decreased (p < 0.05) compared to spores exposed to post-FMT bile acids. This indicates that the combination of intracolonic bile acids prior to FMT induced germination of *C*. *difficile* spores, while the combination of bile acids present after FMT did not.

To test whether the germination response to fecal bile acid combinations varied across *C*. *difficile* strain, we tested spores from nine additional *C*. *difficile* isolates, and the NAP1 strain, that we collected from the household environment of our R-CDI patients. These 10 isolates included representatives from four different NAP types and three distinct toxinotypes (Table 2). Exposure of each of the 10 isolates to colonic bile acid combinations resulted in significant (p<0.05) germination of spores versus post-FMT (Fig 1B, S2 Table). These results indicate that the typical fecal bile acid composition before FMT can induce germination in multiple strains and toxinotypes of *C*. *difficile*, whereas bile acids present after FMT do not.

**Table 2:** Characteristics of *Clostridium difficile* isolates used in this study.

PFGE type (isolate no.)^a^	*tcdC* deletion^b^ (bp)	Binary toxin	Toxinotype (48)
NAP1	18	+	III
NAP2 (i)	0	-	0
NAP2 (ii)	0	-	0
NAP6 (i)	0	-	0
NAP6 (ii)	0	-	0
NAP6 (iii)	0	-	0
NAP7 (i)	39	+	V
NAP7 (ii)	39	-	V
NAP7 (iii)	39	-	V
NAP10	0	-	0

^a^PFGE = Pulsed field gel electrophoresis.

^b^*tcdC* deletion = length of deletion in *tcdC* gene.

**Fig 2:**
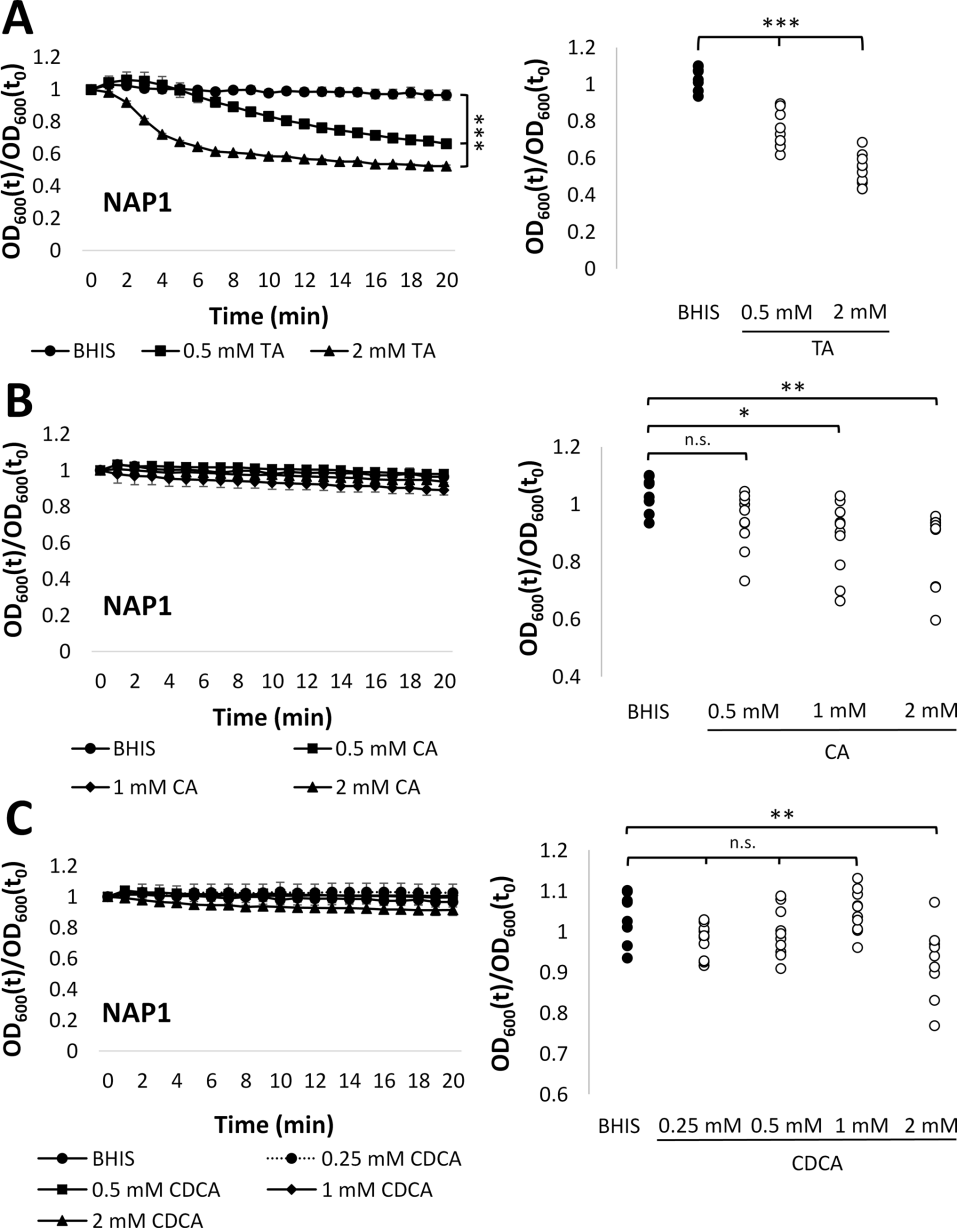
*C*. *difficile* spores germinate in response to primary bile acids. A) (Left Panel) Relative OD_600_ of NAP1 spores exposed to 0.5 mM (square) or 2 mM (triangle) TA versus BHIS alone (circle). (Right Panel) Relative OD_600_ of spores from 10 isolates after 20 min exposure to 0.5 or 2 mM TA vs. BHIS alone. B) (Left Panel) Relative OD_600_ of NAP1 spores exposed to 0.5 mM (square), 1 mM (diamond), or 2 mM (triangle) CA versus BHIS alone (circle). (Right Panel) Relative OD_600_ of spores from 10 isolates after 20 min exposure to 0.5, 1, or 2 mM CA vs. BHIS alone. C) (Left) Relative OD_600_ of NAP1 spores exposed to 0.25 mM (dashed line), 0.5 mM (square), 1 mM (diamond), or 2 mM (triangle) CDCA versus BHIS alone (circle). (Right Panel) Relative OD_600_ of spores from 10 isolates after 20 min exposure to 0.25, 0.5, 1, or 2 mM CDCA vs. BHIS alone. OD_600_(t)/OD_600_(t_0_) = OD_600_ normalized to initial OD_600_ (relative OD_600_). Legends: *** = p < 0.001, ** = p < 0.01; * = p < 0.05, n.s. = non-significant. BHIS = BHI with yeast extract and L-cysteine; TA = taurocholate; CA = cholate; and CDCA = chenodeoxycholic acid. Data represent mean ± SEM.

To investigate what concentration of each bile acid in the pre-FMT combination individually contributed to *C*. *difficile* germination, we examined the effects of a range of concentrations of primary bile acids on spore germination. Taurocholate, which is a known as germinant of *C*. *difficile* spores [23, 28] caused significant (p<0.001) germination (a decrease in relative OD_600_) of spores from all 10 isolates (Fig 2A, S2 Table). While cholate has also been considered a germinant for *C*. *difficile* [23, 28], we found no significant decrease in relative OD_600_ of the NAP1 spores after exposure to cholate. In contrast, the relative OD_600_ of spores from all other 9 isolates decreased significantly following exposure to 1 or 2 mM cholate (p<0.05 and p<0.01, respectively) (Fig 2B). These findings suggest that both taurocholate and cholate induce germination of spores from these clinical isolates of *C*. *difficile*.

In contrast to taurocholate and cholate, chenodeoxycholic acid (CDCA), another major primary bile acid in our pre-FMT combination, is typically found to inhibit *C*. *difficile* germination [24, 25]. Consistent with this, we found that exposure of all 10 isolates to CDCA significantly (p<0.01) inhibited the decrease in relative OD_600_ seen with taurocholate alone (S1 Fig). This result indicates that CDCA inhibits germination of spores from our *C*. *difficile* isolates.

While exposure to CDCA without taurocholate did not decrease the relative OD_600_ of NAP1 spores, 2 mM CDCA caused a significant (p<0.05) decrease in relative OD_600_ for all other 9 isolates (Fig 2C, S2 Table). This suggests that greater concentrations of CDCA can induce germination of spores from some isolates of *C*. *difficile*. Overall, these findings indicate that all three primary bile acids that predominate in pre-FMT feces (taurocholate, cholate, and CDCA) can induce germination of *C*. *difficile* at sufficiently high concentrations. Furthermore, although CDCA can also inhibit taurocholate-mediated germination, the effects of cholate and taurocholate outweigh the effects of CDCA at concentrations similar to those normally found present in feces from pre-FMT patients (Table 1).

**Fig 3:**
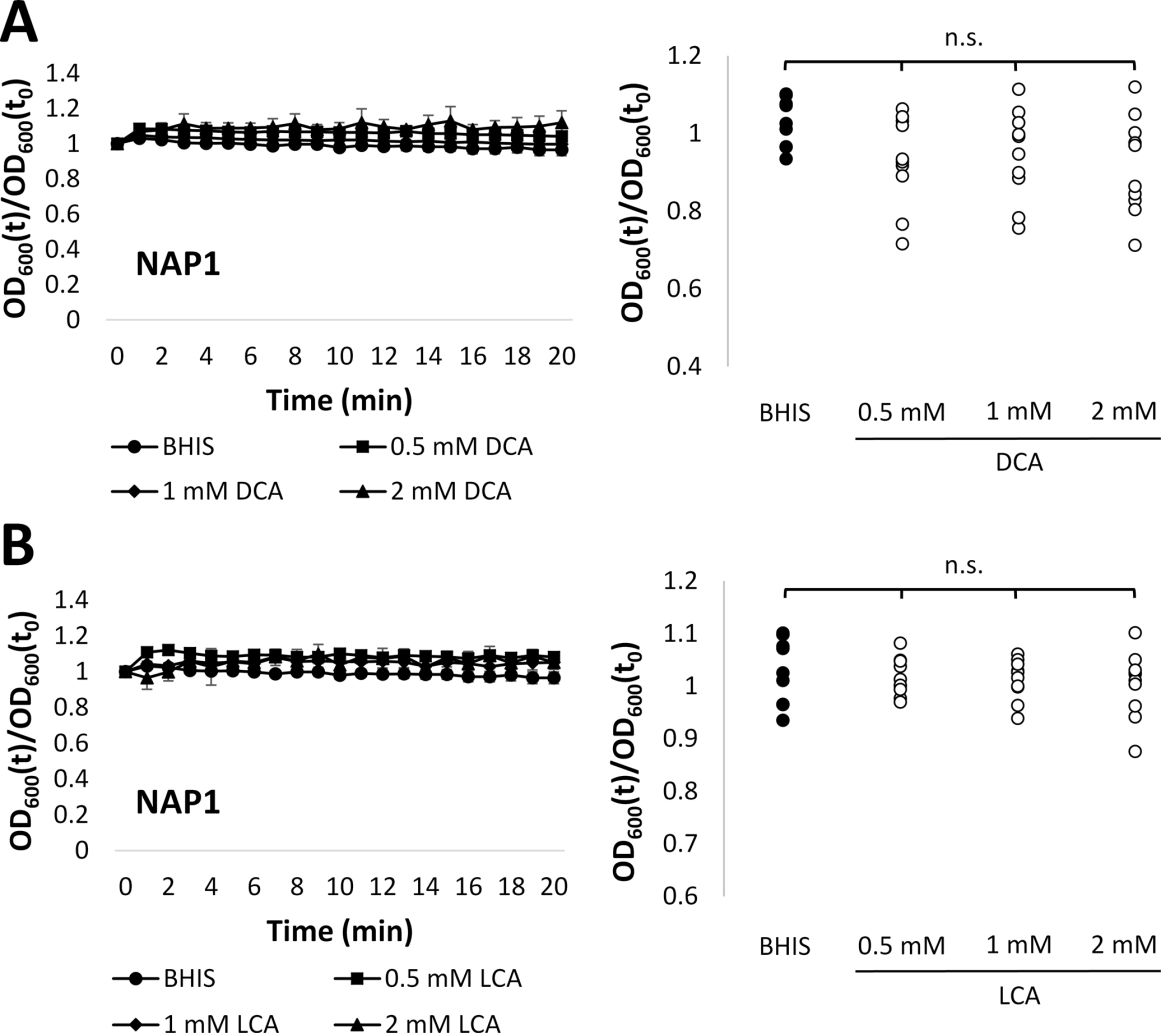
*C*. *difficile* spores do not germinate in response to secondary bile acids. A) (Left Panel) Relative OD_600_ of NAP1 spores exposed to 0.5 mM (square), 1 mM (diamond), or 2 mM (triangle) DCA versus BHIS alone (circle). (Right Panel) Relative OD_600_ of spores from 10 isolates after 20 min exposure to 0.5, 1, or 2 mM DCA vs. BHIS alone. B) (Left Panel) Relative OD_600_ of NAP1 spores exposed to 0.5 mM (square), 1 mM (diamond), or 2 mM (triangle) LCA versus BHIS alone (circle). (Right Panel) Relative OD_600_ of spores from 10 isolates after 20 min exposure to 0.5, 1, or 2 mM LCA vs. BHIS alone. OD_600_(t)/OD_600_(t_0_) = OD_600_ normalized to initial OD_600_ (relative OD_600_). Legend: n.s. = non-significant. BHIS = BHI with yeast extract and L-cysteine; DCA = deoxycholate; and LCA = lithocholic acid. Data represent mean ± SEM.

We tested whether physiological concentrations of deoxycholate led to germination of our isolates by exposing spores to a range of concentrations inclusive of the average concentration of this bile acid in post-FMT patient feces. There was no decrease in relative OD_600_ of spores from the NAP1 isolate alone or of spores from all 10 isolates after exposure to any tested concentration of deoxycholate (Fig 3A, S2 Table). Similarly, no decrease in relative OD_600_ was observed when spores were exposed to any tested concentration of lithocholic acid, the other secondary bile acid present in the post-FMT bile acid combination (Fig 3B, S2 Table). These results indicate that the secondary bile acids deoxycholate and lithocholic acid, which are elevated in post-FMT patient feces, do not induce germination of *C*. *difficile* spores.

Overall, our findings demonstrate that primary bile acids at physiological concentrations typically observed in patient feces prior to FMT can induce germination of *C*. *difficile* spores, likely due to the dominant effects of cholate and taurocholate. Furthermore, the secondary bile acids deoxycholate and lithocholic acid do not induce germination of *C*. *difficile* at the concentrations found in patient feces after FMT.

### Fecal bile acids present after FMT strongly inhibit vegetative growth of *C*. *difficile*

**Fig 4:**
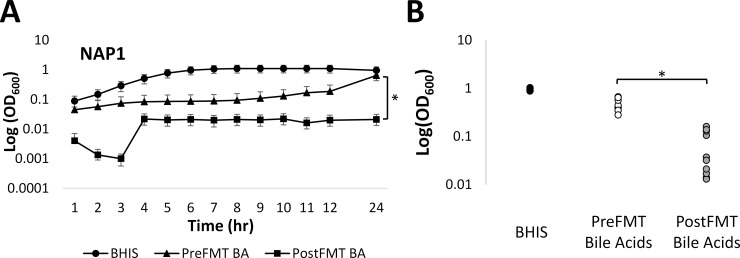
Growth of vegetative *C*. *difficile* is inhibited by post-FMT bile acids. A) Hourly OD_600_ measurements for NAP1 cells in BHIS alone (circle), BHIS with pre-FMT bile acids (triangle), and BHIS with post-FMT bile acids (square). B) OD_600_ at 24 h for 10 isolates. Legend: BA = bile acids; and * = p <0.01. Data represent mean ± SEM.

We also investigated whether pre- and post-FMT bile acid combinations affected vegetative growth of *C*. *difficile*. Overnight cultures of all 10 isolates were grown with taurocholate to induce germination, and the resulting vegetative cells were inoculated into BHIS containing pre- or post-FMT bile acid combinations. Results in Fig 4A and S2 Fig show that the growth of cells in BHIS containing either pre-FMT bile acids or post-FMT bile acids was delayed relative to cells grown in BHIS alone. However, in 9 of 10 isolates, the OD_600_ of cells after 24 h in medium with post-FMT bile acids was significantly (p<0.05) lower than cells grown with pre-FMT bile acids. This suggests that secondary bile acids present in feces of post-FMT patient also inhibited vegetative growth of *C*. *difficile* compared to bile acids found in patient feces before FMT. This effect was not likely due to a decrease in pH from the addition of bile acids, since the pH of BHIS alone was not significantly different from the pH of BHIS containing either combination of bile acids (data not shown).

### Variation in germination response to bile acids may be explained by variations in the *C*. *difficile* germinant receptor

While our findings indicated that pre-FMT bile acids induced germination of *C*. *difficile* spores across a range of geno- and toxino-types, it was previously reported that there is differential response of spores from different isolates to bile acids [27]. We therefore investigated whether there was variation in the germination response of spores from five strains representative of our 10 isolates to our pre-FMT bile acid combinations. Results in S3 Fig show that while all strains germinated in response to pre-FMT bile acids, compared to those present in feces of post-FMT bile patients, this response varied by strain genotype, and was most profound in NAP7 isolates.

### Variation in germination receptor

**Fig 5:**
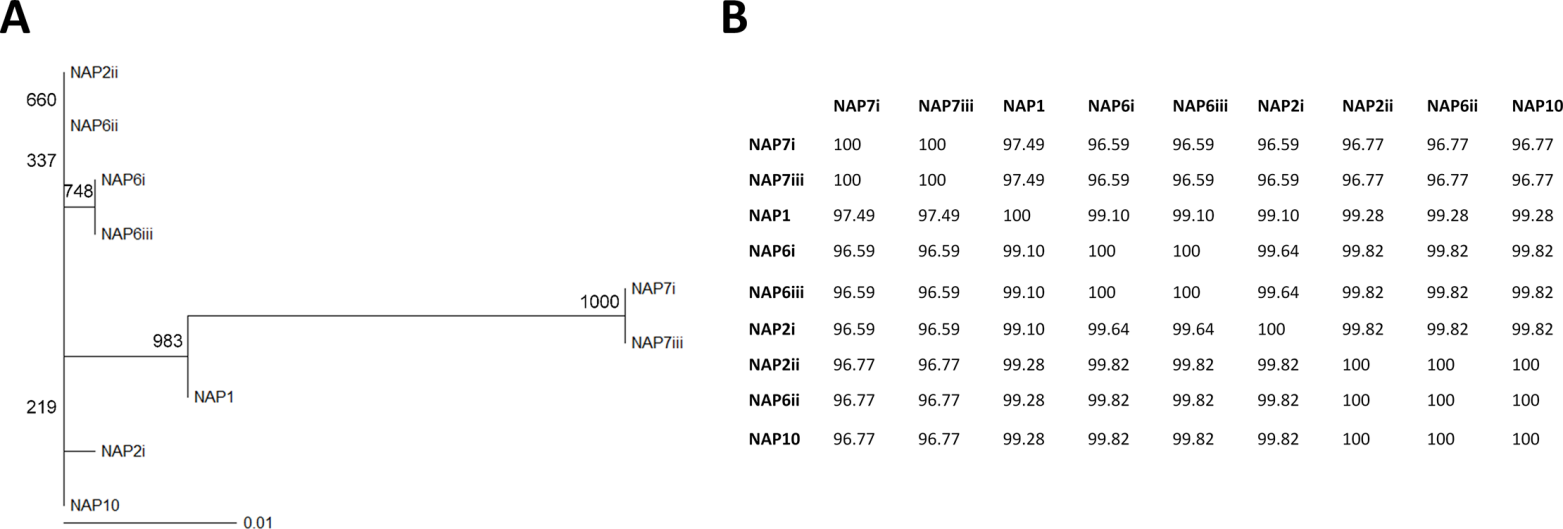
CspC sequences primarily cluster by PFGE type. A) Boot-strapped phylogenetic tree generated by UPGMA method. Boot-strap values are indicated at each node. B) Percent identity matrix for CspC sequences. Roman numerals indicate isotype number within each PFGE type. Legend: NAP = North American pulsed-field gel electrophoresis (PFGE) type.

While little is known about the genetics and mechanisms of *C*. *difficile* germination, recent work has revealed that CspC, an analog of the serine protease involved in germination in *Clostridium perfringens*, is a germinant receptor which senses bile acids in *C*. *difficile* [37]. We therefore hypothesized that some variation in bile acid-induced germination of spores from our isolates might be due to variations in this receptor. To test this hypothesis, we generated two sets of PCR primers, which targeted the published *cspC* gene sequence in *C*. *difficile* [37] and successfully sequenced the *cspC* gene from nine of our isolates (S1 Table). Alignment of the protein sequences translated from the sequenced *cspC* genes revealed that while all sequences were highly similar, CspC sequences tended to cluster by strain (Fig 5, S4 Fig). While sequences from the two NAP7 isolates were identical, all other sequences were more similar to each other (≥ 99.1% r similarity), than they were to NAP7 sequences (96.6 to 97.5% similarity) (Fig 5B). We identified a total of 14 amino acid substitutions unique to the NAP7 isolates compared to all other isolates (Table 3), of which 7 were conservative, 3 were semi-conservative, and 4 were non-conservative amino acid substitutions. These findings suggest that mutations in the *C*. *difficile* germinant receptor may be involved in differences in germination of spores in response to bile acids present in the feces of R-CDI patients. This, however, needs to be verified with more detailed studies.

**Table 3:** Amino acid substitutions in CspC protein sequences of NAP7 isolates.

Position	Amino acid in most isolates	Amino acid in NAP7 isolates	Type of substitution
47	Leu	Ile	Conservative
59	Glu	Ala	Non-conservative
73	Asn	Asp	Conservative
83	Glu	Gly	Non-conservative
85	Asp	Glu	Conservative
179	Val	Ala	Semi-conservative
184	Ile	Val	Conservative
187	Gln	Lys	Conservative
206	Thr	Ile	Non-conservative
207	Asp	Gly	Semi-conservative
237	Lys	Arg	Conservative
250	Ser	Gly	Semi-conservative
338	Ser	Ala	Conservative
384	Ile	Thr	Non-conservative

## Discussion

Although fecal microbiota transplantation (FMT) is a proven highly effective treatment for R-CDI [11–13], its acceptance into mainstream medicine requires further development and regulatory approval [38]. Furthermore, some patients may not benefit from FMT, including those who require frequent courses of antibiotics for other infections. Therefore, there remains a need to develop non-antibiotic approaches to therapy for R-CDI. A mechanistic understanding of FMT has the potential to enable more rational development of such therapeutics.

Microbial metabolism of intracolonic bile acids provides a link between the transplantation of a healthy microbiome and lasting recovery from CDI. The impact of bile acids on the *C*. *difficile* lifecycle has long been recognized. For example, taurocholate is routinely used in *C*. *difficile* culture media [28, 29], and both taurocholate and cholate are known to induce spore germination of the bacterium [23]. In contrast, lithocholic acid, one of the dominant colonic secondary bile acids produced from primary bile acids via bacterial metabolism, is a known inhibitor of *C*. *difficile* germination [24, 25]. The impact of bile acids on pathogenicity of *C*. *difficile* is further supported by animal models of the infection. The abundance of intracolonic secondary bile acids is markedly decreased in mice treated with antibiotics and susceptible to CDI and is significantly greater in mice resistant to CDI [26, 39–41]. These findings suggest that the types of bile acids present in the colon and the ability of *C*. *difficile* to respond to those compounds may play an important role in this infection.

We previously showed that secondary bile acids are absent in the feces of patients with R-CDI syndrome, whereas primary bile acids are abundant [22]. FMT promptly normalizes the fecal bile acid composition, decreasing primary bile acid concentrations and increasing secondary bile acids to levels found in healthy individuals. Consequently, we hypothesized that suppression of secondary bile acid metabolism in the colon by antibiotics creates an environment that is favorable for *C*. *difficile* germination and growth, and that restoration of normal secondary bile acid metabolism contributes to the clinical success of FMT in the treatment of R-CDI.

We tested these hypotheses here by measuring *C*. *difficile* spore germination and vegetative growth in the presence of mixtures of bile acids at concentrations found in pre-FMT and post-FMT feces of patients treated for R-CDI. Our results indicated that both germination and growth were inhibited in all tested clinical isolates of *C*. *difficile* in the presence of post-FMT fecal bile acids (secondary bile acids) compared to pre-FMT bile acids (primary bile acids). However, recent work suggests that a model wherein primary bile acids promote *C*. *difficile* germination and secondary bile acids are inhibitory might be overly simplistic. One of the major primary bile acids in humans, chenodeoxycholic acid (CDCA), can prevent taurocholate-mediated spore germination [23, 24]. We confirmed these findings, although as previously reported we also found that high (non-physiological) concentrations of CDCA (2 mM) cause some strains of *C*. *difficile* to germinate [27]. Importantly, however, CDCA is absorbed in the colon with far greater efficiency than cholate [42], which diminishes its intraluminal impact relative to cholate derivatives. As we have shown, the resulting low CDCA concentration in pre-FMT feces cannot fully inhibit taurocholate- and cholate-induced *C*. *difficile* germination.

Furthermore, it is not clear that secondary bile acids do not promote *C*. *difficile* germination. Deoxycholate, which is abundant in feces from healthy donors and post-FMT patient, has been shown to be a germinant for *C*. *difficile* [23, 28]. Our results, however, suggest that many *C*. *difficile* strains do not germinate in response to physiologically relevant concentrations of deoxycholate (0.5 to 2 mM) as measured in post-FMT feces. Furthermore, compared to taurocholate, even greater concentrations of deoxycholate induce only modest germination [23, 39]. Lithocholic acid, which is also elevated in post-FMT and donor feces, is not known to cause germination [25], in agreement with our present findings. Overall, our results suggest the combination of these two bile acids do not induce *C*. *difficile* germination, and emphasizes the importance of assessing the impact of combinations of bile acids which reflect the colonic environment in human patients, rather than individual bile acids.

One limitation of our study was the use of measurements of bile acids in feces, rather than true intracolonic concentrations. The process of transforming primary to secondary bile acids by bacteria, via 7α-dehydroxylation, is thought to be exclusively performed in the colon [43]. Thus, it is likely that concentrations of primary bile acids measured in the feces may underestimate those in the proximal colon, and conversely, concentrations of secondary bile acids may be overestimated. However, our results also demonstrated that post-FMT bile acids inhibit vegetative growth of *C*. *difficile*, suggesting that even if some germination can occur in healthy or post-FMT individuals, proliferation of the bacteria would be limited.

Importantly, our findings were consistent across all 10 tested *C*. *difficile* isolates, including a representative of the NAP1 strain, which is considered hypervirulent and responsible for several epidemic outbreaks of CDI [36]. Since the germination response to CDCA varies among *C*. *difficile* strains [27], it is necessary to investigate the response of a range of isolates to these bile acids. All of our tested *C*. *difficile* strains were clinical isolates from R-CDI patients who were treated with FMT, likely representing the strains responsible for disease in these patients. Therefore, our findings are likely to be directly applicable to future R-CDI patients.

Although these findings were overall consistent across our *C*. *difficile* isolates, we also found that the magnitude of response varied by PFGE type. In particular, isolates from the NAP7 PFGE type demonstrated a notably stronger response to the pre-FMT bile acid combination. This finding is not unexpected, given previous demonstration of variation in *C*. *difficile* germination response to bile acids across a variety of strains and isolates [27].

To explain the variation we found here, we sequenced the recently identified bile acid receptor, the serine protease CspC, from nine of our isolates [37]. Our results indicated that although CspC sequence is highly similar across isolates, the CspC sequence of NAP7 isolates is somewhat more distinct, matching well with our germination results. Although the catalytic domain of *C*. *difficile* CspC is absent [37], limiting our ability to interpret the significance of mutations in NAP7 CspC compared to our other isolates, the presence of several non-conservative mutations suggests potential sites of interest which could be investigated further. Notably, NAP7 (ribotype 078), like NAP1, is known to be a hypervirulent strain of *C*. *difficile* and is associated with an increased mortality rate [44]. It is therefore possible that this increased sensitivity to bile acid germinants, related to mutations in the germinant receptor, is a mechanism of increased virulence in this strain. In addition to fully exploring these mutations, sequencing of the CspC protein from additional isolates, from both NAP7 and other PFGE types, will be necessary to fully understand the significance of these results.

Clinical success of FMT provides an opportunity to gain mechanistic insight into how indigenous microbial communities limit *C*. *difficile*. Mechanistic understanding is critical for rational design of new therapeutics, which may be easier to regulate than FMT and may benefit patients who do not qualify for FMT. Our results here provide strong support to the hypothesis that the observed shift in bile acid composition following FMT plays an important role in the therapeutic efficacy of the procedure.

### Ethics Statement

The study was approved by the University of Minnesota Institutional Review Board (IRB). Collection of household *C*. *difficile* isolates was performed under IRB 1112M07323. The study of fecal specimens was performed under IRB 0901M56962. All human subjects provided written informed consent prior to participation in the study.

## Supporting Information

S1 Fig CDCA inhibits taurocholate-mediated germination of *C*. *difficile*. A) Relative OD_600_ of NAP1 spores exposed to 0.25 mM (dashed line; circle), 0.5 mM (square), 1 mM (diamond), or 2 mM (triangle) CDCA and 2 mM TA versus TA alone (circle) in BHIS. B) Relative OD_600_ of spores from 10 isolates after 20 min exposure to 0.25, 0.5, 1, or 2 mM CDCA and 2 mM TA vs. TA alone in BHIS. OD_600_(t)/OD_600_(t_0_) = OD_600_ normalized to initial OD_600_ (relative OD_600_). Legend: * = p <0.01. BHIS = BHI with yeast extract and L-cysteine; TA = taurocholate; and CDCA = chenodeoxycholic acid. Experiments performed in triplicate under anaerobic conditions. Data represent mean ± SEM.
(TIF)

S2 Fig Growth of vegetative *C*. *difficile* isolates in the presence of fecal bile acids. Hourly OD_600_ measurements for cells in BHIS alone (circle), BHIS with PreFMT bile acids (triangle), and BHIS with PostFMT bile acids (square). A-B) NAP2 isolates. **C**) NAP10 isolate. D-F) NAP6 isolates. G-I) NAP7 isolates. Legend: * = p<0.01; and n.s. = non-significant. Experiments were performed in triplicate. Data represent mean ± SEM.
(TIF)

S3 Fig Germination of *C*. *difficile* spores varies by strain. Relative OD_600_ of spores from the five PFGE types used in this study when exposed to BHIS alone (black circles), BHIS with bile acids at concentrations found in pre-FMT patient feces (PreFMT; grey circles), or BHIS with bile acids at concentrations found in post-FMT patient feces (PostFMT; white circles) for 20 min. OD_600_(t)/OD_600_(t_0_) = OD_600_ normalized to initial OD_600_ (relative OD_600_).
(TIF)

S4 Fig Alignment of *C*. *difficile* CspC protein sequences. Protein sequences from 9 isolates. Roman numerals represent isolate number within PFGE type. Highlighted loci have previously been reported to be essential for germination [37]. Legend: * = conserved residue;: = conservative mutation;. = semi-conservative mutation; blank = non-conservative mutation; and NAP = North American pulsed-field gel electrophoresis (PFGE) type.
(TIF)

S1 Table *C*. *difficile cspC* PCR primers used in this study. (DOCX)

S2 Table Mean relative OD_600_ of spores from 10 isolates after 20 min exposure to bile acids. (DOCX)

## References

[1] Kelly CP, LaMont JT. Clostridium difficile—more difficult than ever. N Engl J Med. 2008;359(18):1932–40. 10.1056/NEJMra0707500 . 18971494

[2] Miller BA, Chen LF, Sexton DJ, Anderson DJ. Comparison of the burdens of hospital-onset, healthcare facility-associated Clostridium difficile Infection and of healthcare-associated infection due to methicillin-resistant Staphylococcus aureus in community hospitals. Infect Control Hosp Epidemiol. 2011;32(4):387–90. 10.1086/659156 . 21460491

[3] Ananthakrishnan AN. Clostridium difficile infection: epidemiology, risk factors and management. Nature reviews Gastroenterology & hepatology. 2011;8(1):17–26. 10.1038/nrgastro.2010.190 . 21119612

[4] Lessa FC, Gould CV, McDonald LC. Current status of Clostridium difficile infection epidemiology. Clin Infect Dis. 2012;55 Suppl 2:S65–70. 10.1093/cid/cis319 22752867 PMC3388017

[5] Khanna S, Pardi DS, Aronson SL, Kammer PP, Orenstein R, St Sauver JL, et al The epidemiology of community-acquired Clostridium difficile infection: a population-based study. Am J Gastroenterol. 2012;107(1):89–95. 10.1038/ajg.2011.398 22108454 PMC3273904

[6] Chitnis AS, Holzbauer SM, Belflower RM, Winston LG, Bamberg WM, Lyons C, et al Epidemiology of community-associated Clostridium difficile infection, 2009 through 2011. JAMA internal medicine. 2013;173(14):1359–67. 10.1001/jamainternmed.2013.7056 . 23780507 PMC11931991

[7] Lessa FC, Mu Y, Bamberg WM, Beldavs ZG, Dumyati GK, Dunn JR, et al Burden of Clostridium difficile infection in the United States. N Engl J Med. 2015;372(9):825–34. 10.1056/NEJMoa1408913 . 25714160 PMC10966662

[8] Louie TJ, Miller MA, Mullane KM, Weiss K, Lentnek A, Golan Y, et al Fidaxomicin versus vancomycin for Clostridium difficile infection. N Engl J Med. 2011;364(5):422–31. 10.1056/NEJMoa0910812 . 21288078

[9] Borody TJ, Khoruts A. Fecal microbiota transplantation and emerging applications. Nature reviews Gastroenterology & hepatology. 2012;9(2):88–96. 10.1038/nrgastro.2011.244 . 22183182

[10] Surawicz CM, Alexander J. Treatment of refractory and recurrent Clostridium difficile infection. Nature reviews Gastroenterology & hepatology. 2011;8(6):330–9. 10.1038/nrgastro.2011.59 . 21502971

[11] Gough E, Shaikh H, Manges AR. Systematic review of intestinal microbiota transplantation (fecal bacteriotherapy) for recurrent Clostridium difficile infection. Clin Infect Dis. 2011;53(10):994–1002. 10.1093/cid/cir632 . 22002980

[12] Hamilton MJ, Weingarden AR, Sadowsky MJ, Khoruts A. Standardized frozen preparation for transplantation of fecal microbiota for recurrent Clostridium difficile infection. Am J Gastroenterol. 2012;107(5):761–7. 10.1038/ajg.2011.482 . 22290405

[13] van Nood E, Vrieze A, Nieuwdorp M, Fuentes S, Zoetendal EG, de Vos WM, et al Duodenal infusion of donor feces for recurrent Clostridium difficile. N Engl J Med. 2013;368(5):407–15. 10.1056/NEJMoa1205037 . 23323867

[14] Youngster I, Sauk J, Pindar C, Wilson RG, Kaplan JL, Smith MB, et al Fecal microbiota transplant for relapsing Clostridium difficile infection using a frozen inoculum from unrelated donors: a randomized, open-label, controlled pilot study. Clin Infect Dis. 2014;58(11):1515–22. 10.1093/cid/ciu135 24762631 PMC4017893

[15] Khoruts A, Dicksved J, Jansson JK, Sadowsky MJ. Changes in the composition of the human fecal microbiome after bacteriotherapy for recurrent Clostridium difficile-associated diarrhea. J Clin Gastroenterol. 2010;44(5):354–60. 10.1097/MCG.0b013e3181c87e02 . 20048681

[16] Shahinas D, Silverman M, Sittler T, Chiu C, Kim P, Allen-Vercoe E, et al Toward an understanding of changes in diversity associated with fecal microbiome transplantation based on 16S rRNA gene deep sequencing. mBio. 2012;3(5). 10.1128/mBio.00338-12 23093385 PMC3482503

[17] Hamilton MJ, Weingarden AR, Unno T, Khoruts A, Sadowsky MJ. High-throughput DNA sequence analysis reveals stable engraftment of gut microbiota following transplantation of previously frozen fecal bacteria. Gut microbes. 2013;4(2):125–35. 10.4161/gmic.23571 23333862 PMC3595072

[18] Seekatz AM, Aas J, Gessert CE, Rubin TA, Saman DM, Bakken JS, et al Recovery of the gut microbiome following fecal microbiota transplantation. mBio. 2014;5(3):e00893–14. 10.1128/mBio.00893-14 24939885 PMC4068257

[19] Fuentes S, van Nood E, Tims S, Heikamp-de Jong I, ter Braak CJ, Keller JJ, et al Reset of a critically disturbed microbial ecosystem: faecal transplant in recurrent Clostridium difficile infection. The ISME journal. 2014;8(8):1621–33. 10.1038/ismej.2014.13 . 24577353 PMC4817604

[20] Shankar V, Hamilton MJ, Khoruts A, Kilburn A, Unno T, Paliy O, et al Species and genus level resolution analysis of gut microbiota in Clostridium difficile patients following fecal microbiota transplantation. Microbiome. 2014;2:13 10.1186/2049-2618-2-13 24855561 PMC4030581

[21] Weingarden A, Gonzalez A, Vazquez-Baeza Y, Weiss S, Humphry G, Berg-Lyons D, et al Dynamic changes in short- and long-term bacterial composition following fecal microbiota transplantation for recurrent Clostridium difficile infection. Microbiome. 2015;3:10 10.1186/s40168-015-0070-0 25825673 PMC4378022

[22] Weingarden AR, Chen C, Bobr A, Yao D, Lu Y, Nelson VM, et al Microbiota transplantation restores normal fecal bile acid composition in recurrent Clostridium difficile infection. Am J Physiol Gastrointest Liver Physiol. 2014;306(4):G310–9. 10.1152/ajpgi.00282.2013 24284963 PMC3920123

[23] Sorg JA, Sonenshein AL. Bile salts and glycine as cogerminants for Clostridium difficile spores. J Bacteriol. 2008;190(7):2505–12. 10.1128/JB.01765-07 18245298 PMC2293200

[24] Sorg JA, Sonenshein AL. Chenodeoxycholate is an inhibitor of Clostridium difficile spore germination. J Bacteriol. 2009;191(3):1115–7. 10.1128/JB.01260-08 19060152 PMC2632082

[25] Sorg JA, Sonenshein AL. Inhibiting the initiation of Clostridium difficile spore germination using analogs of chenodeoxycholic acid, a bile acid. J Bacteriol. 2010;192(19):4983–90. 10.1128/JB.00610-10 20675492 PMC2944524

[26] Giel JL, Sorg JA, Sonenshein AL, Zhu J. Metabolism of bile salts in mice influences spore germination in Clostridium difficile. PLoS One. 2010;5(1):e8740 10.1371/journal.pone.0008740 20090901 PMC2806926

[27] Heeg D, Burns DA, Cartman ST, Minton NP. Spores of Clostridium difficile clinical isolates display a diverse germination response to bile salts. PLoS One. 2012;7(2):e32381 10.1371/journal.pone.0032381 22384234 PMC3285209

[28] Wilson KH. Efficiency of various bile salt preparations for stimulation of Clostridium difficile spore germination. J Clin Microbiol. 1983;18(4):1017–9. 6630458 10.1128/jcm.18.4.1017-1019.1983 PMC270959

[29] Arroyo LG, Rousseau J, Willey BM, Low DE, Staempfli H, McGeer A, et al Use of a selective enrichment broth to recover Clostridium difficile from stool swabs stored under different conditions. J Clin Microbiol. 2005;43(10):5341–3. 10.1128/JCM.43.10.5341-5343.2005 16208013 PMC1248507

[30] Braun V, Hundsberger T, Leukel P, Sauerborn M, von Eichel-Streiber C. Definition of the single integration site of the pathogenicity locus in Clostridium difficile. Gene. 1996;181(1–2):29–38. . 8973304 10.1016/s0378-1119(96)00398-8

[31] Rupnik M, Avesani V, Janc M, von Eichel-Streiber C, Delmee M. A novel toxinotyping scheme and correlation of toxinotypes with serogroups of Clostridium difficile isolates. J Clin Microbiol. 1998;36(8):2240–7. 9665999 10.1128/jcm.36.8.2240-2247.1998 PMC105025

[32] Spigaglia P, Mastrantonio P. Molecular analysis of the pathogenicity locus and polymorphism in the putative negative regulator of toxin production (TcdC) among Clostridium difficile clinical isolates. J Clin Microbiol. 2002;40(9):3470–5. 12202595 10.1128/JCM.40.9.3470-3475.2002 PMC130716

[33] Killgore G, Thompson A, Johnson S, Brazier J, Kuijper E, Pepin J, et al Comparison of seven techniques for typing international epidemic strains of Clostridium difficile: restriction endonuclease analysis, pulsed-field gel electrophoresis, PCR-ribotyping, multilocus sequence typing, multilocus variable-number tandem-repeat analysis, amplified fragment length polymorphism, and surface layer protein A gene sequence typing. J Clin Microbiol. 2008;46(2):431–7. 10.1128/JCM.01484-07 18039796 PMC2238077

[34] Larkin MA, Blackshields G, Brown NP, Chenna R, McGettigan PA, McWilliam H, et al Clustal W and Clustal X version 2.0. Bioinformatics. 2007;23(21):2947–8. 10.1093/bioinformatics/btm404 . 17846036

[35] Moir A, Smith DA. The genetics of bacterial spore germination. Annu Rev Microbiol. 1990;44:531–53. 10.1146/annurev.mi.44.100190.002531 . 2252393

[36] McDonald LC, Killgore GE, Thompson A, Owens RC Jr, Kazakova SV, Sambol SP, et al An epidemic, toxin gene-variant strain of Clostridium difficile. N Engl J Med. 2005;353(23):2433–41. 10.1056/NEJMoa051590 . 16322603

[37] Francis MB, Allen CA, Shrestha R, Sorg JA. Bile acid recognition by the Clostridium difficile germinant receptor, CspC, is important for establishing infection. PLoS Pathog. 2013;9(5):e1003356 10.1371/journal.ppat.1003356 23675301 PMC3649964

[38] Khoruts A, Sadowsky MJ, Hamilton MJ. Development of fecal microbiota transplantation suitable for mainstream medicine. Clin Gastroenterol Hepatol. 2014 10.1016/j.cgh.2014.11.014 . 25460566

[39] Theriot CM, Koenigsknecht MJ, Carlson PE Jr, Hatton GE, Nelson AM, Li B, et al Antibiotic-induced shifts in the mouse gut microbiome and metabolome increase susceptibility to Clostridium difficile infection. Nature communications. 2014;5:3114 10.1038/ncomms4114 24445449 PMC3950275

[40] Buffie CG, Bucci V, Stein RR, McKenney PT, Ling L, Gobourne A, et al Precision microbiome reconstitution restores bile acid mediated resistance to Clostridium difficile. Nature. 2014 10.1038/nature13828 . 25337874 PMC4354891

[41] Koenigsknecht MJ, Theriot CM, Bergin IL, Schumacher CA, Schloss PD, Young VB. Dynamics and Establishment of Clostridium difficile Infection in the Murine Gastrointestinal Tract. Infect Immun. 2014 10.1128/IAI.02768-14 . 25534943 PMC4333439

[42] Mekhjian HS, Phillips SF, Hofmann AF. Colonic absorption of unconjugated bile acids: perfusion studies in man. Dig Dis Sci. 1979;24(7):545–50. . 456241 10.1007/BF01489324

[43] Ridlon JM, Kang DJ, Hylemon PB. Bile salt biotransformations by human intestinal bacteria. J Lipid Res. 2006;47(2):241–59. 10.1194/jlr.R500013-JLR200 . 16299351

[44] Walker AS, Eyre DW, Wyllie DH, Dingle KE, Griffiths D, Shine B, et al Relationship between bacterial strain type, host biomarkers, and mortality in Clostridium difficile infection. Clin Infect Dis. 2013;56(11):1589–600. 10.1093/cid/cit127 23463640 PMC3641870

