# Supplementary figures and images for: Changes in Colonic Bile Acid Composition following Fecal Microbiota Transplantation Are Sufficient to Control Clostridium difficile Germination and Growth
Source: PLoS One. 2016 Jan 20;11(1):e0147210. doi: 10.1371/journal.pone.0147210 (PMC4720481; doi:10.1371/journal.pone.0147210)

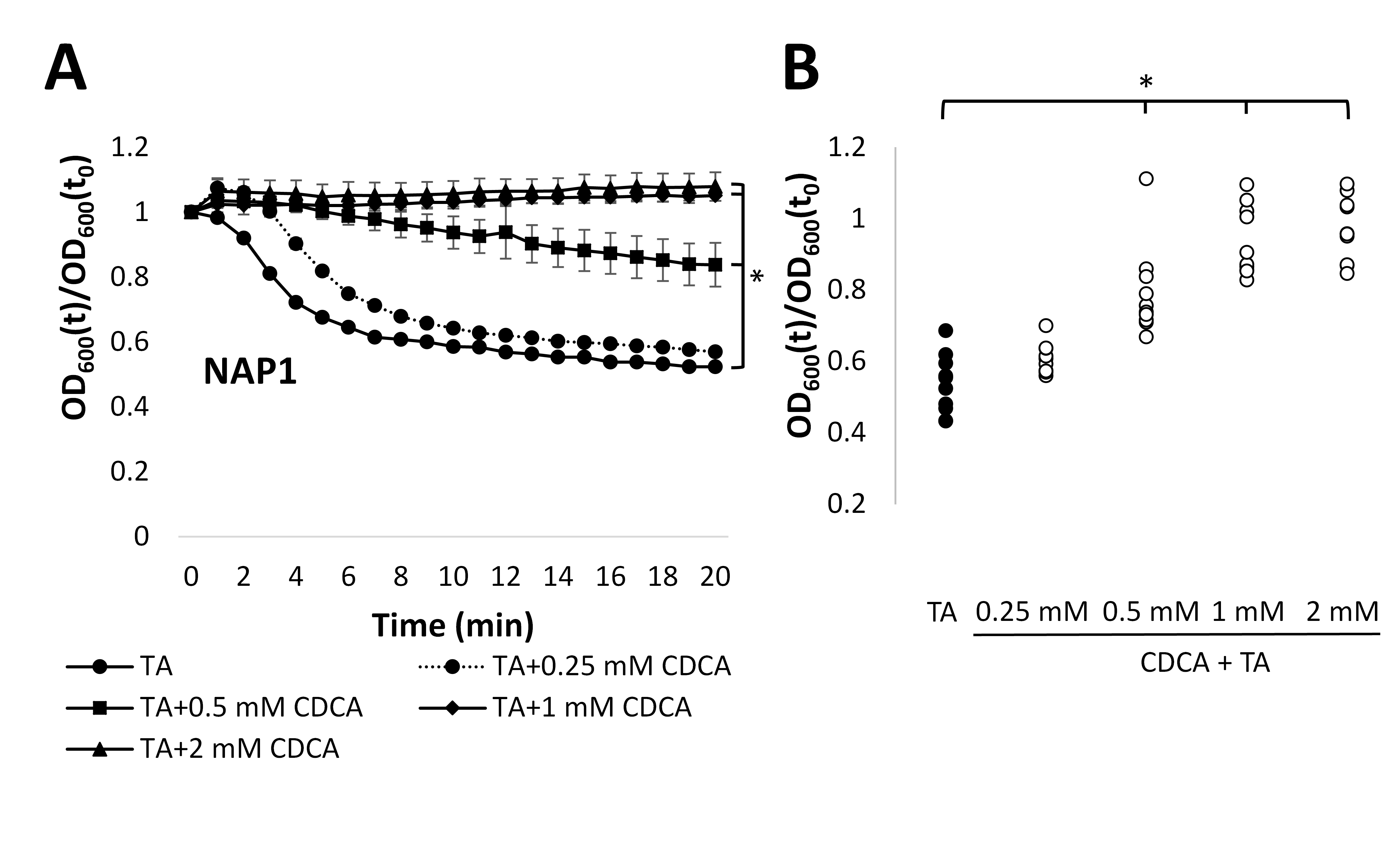

Supplement: S1 Fig — A) Relative OD600 of NAP1 spores exposed to 0.25 mM (dashed line; circle), 0.5 mM (square), 1 mM (diamond), or 2 mM (triangle) CDCA and 2 mM TA versus TA alone (circle) in BHIS. B) Relative OD600 of spores from 10 isolates after 20 min exposure to 0.25, 0.5, 1, or 2 mM CDCA and 2 mM TA vs. TA alone in BHIS. OD600(t)/OD600(t0) = OD600 normalized to initial OD600 (relative OD600). Legend: * = p <0.01. BHIS = BHI with yeast extract and L-cysteine; TA = taurocholate; and CDCA = chenodeoxycholic acid. Experiments performed in triplicate under anaerobic conditions. Data represent mean ± SEM. (TIF) [file pone.0147210.s001.tif]

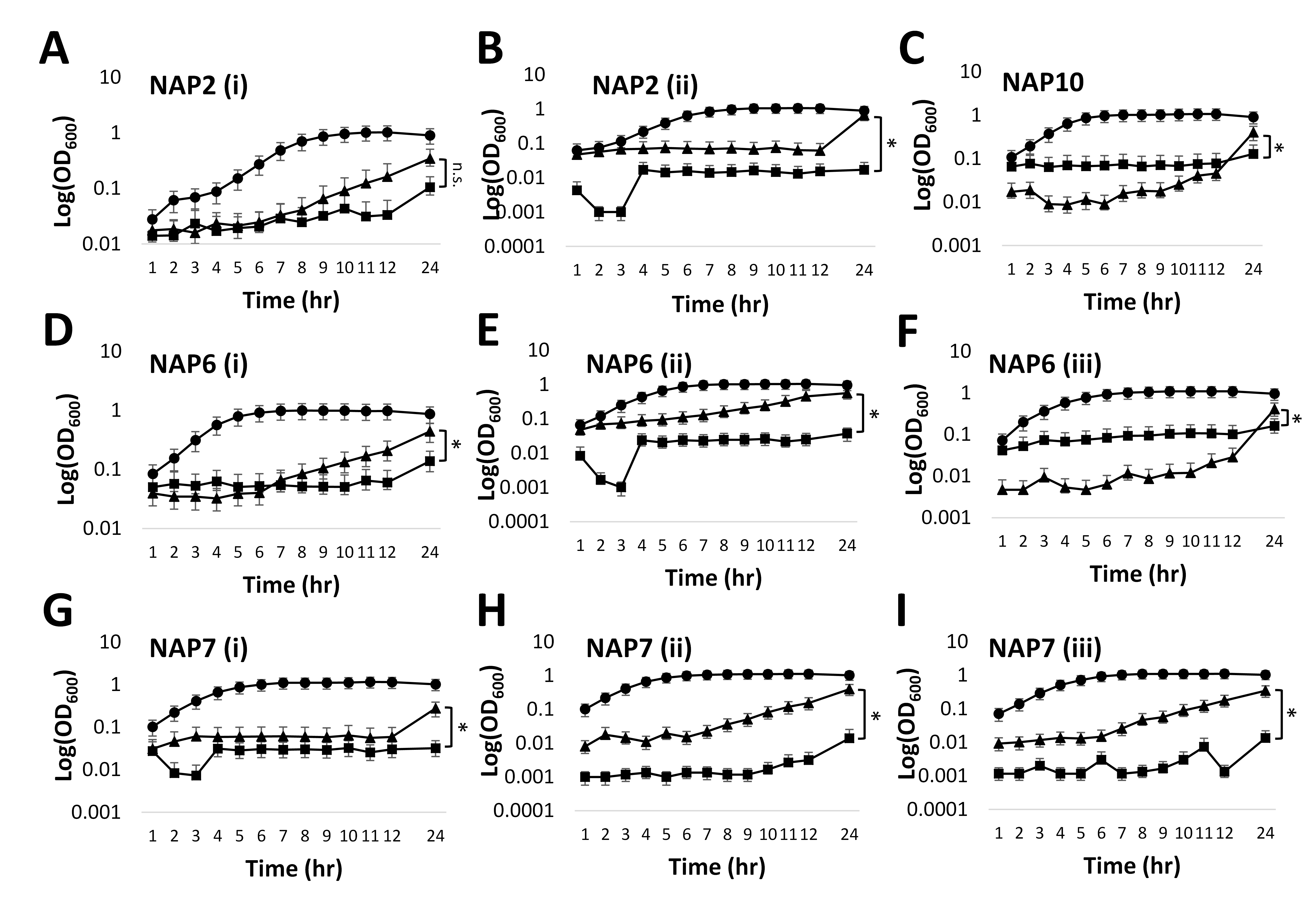

Supplement: S2 Fig — Hourly OD600 measurements for cells in BHIS alone (circle), BHIS with PreFMT bile acids (triangle), and BHIS with PostFMT bile acids (square). A-B) NAP2 isolates. C) NAP10 isolate. D-F) NAP6 isolates. G-I) NAP7 isolates. Legend: * = p<0.01; and n.s. = non-significant. Experiments were performed in triplicate. Data represent mean ± SEM. (TIF) [file pone.0147210.s002.tif]

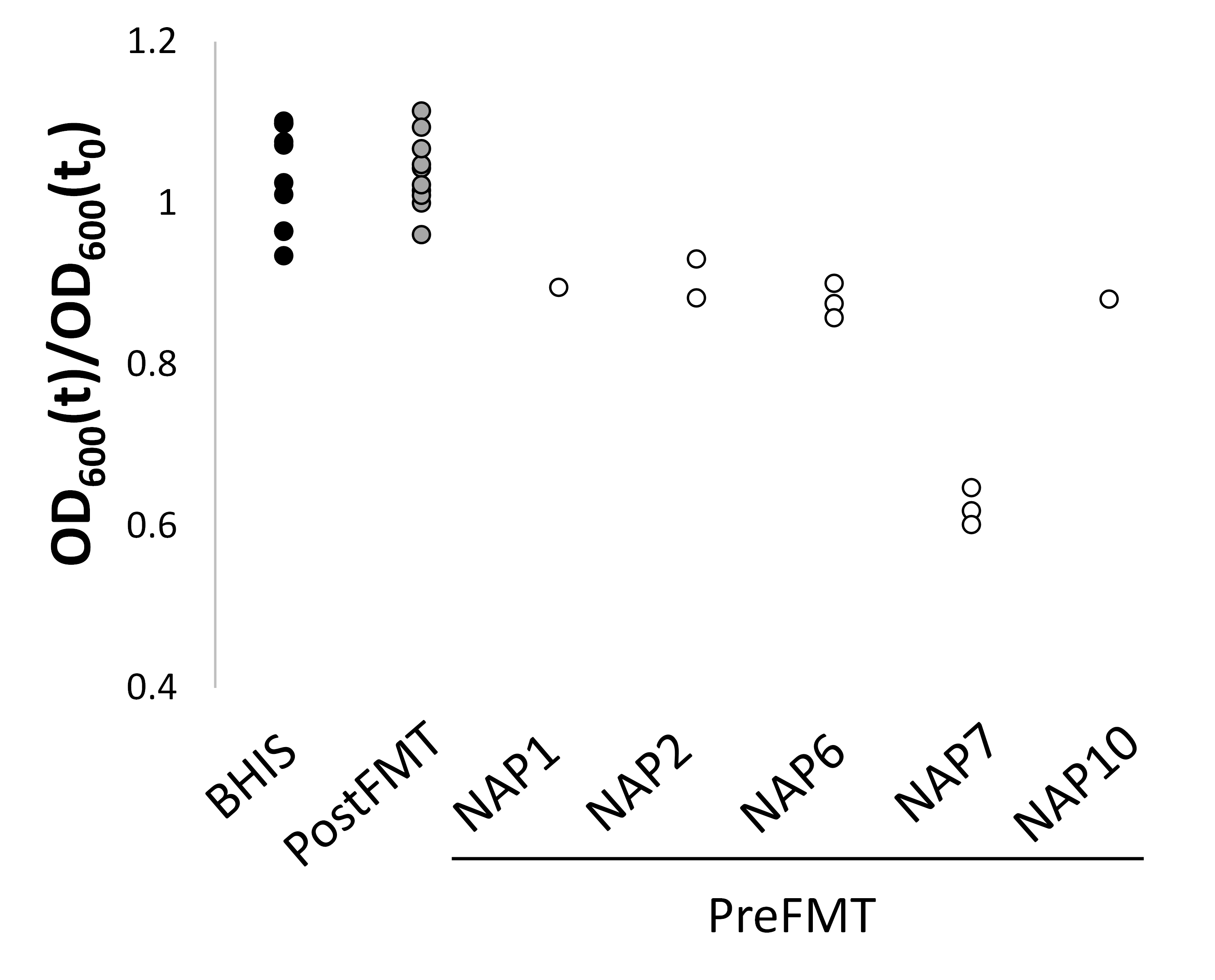

Supplement: S3 Fig — Relative OD600 of spores from the five PFGE types used in this study when exposed to BHIS alone (black circles), BHIS with bile acids at concentrations found in pre-FMT patient feces (PreFMT; grey circles), or BHIS with bile acids at concentrations found in post-FMT patient feces (PostFMT; white circles) for 20 min. OD600(t)/OD600(t0) = OD600 normalized to initial OD600 (relative OD600). (TIF) [file pone.0147210.s003.tif]

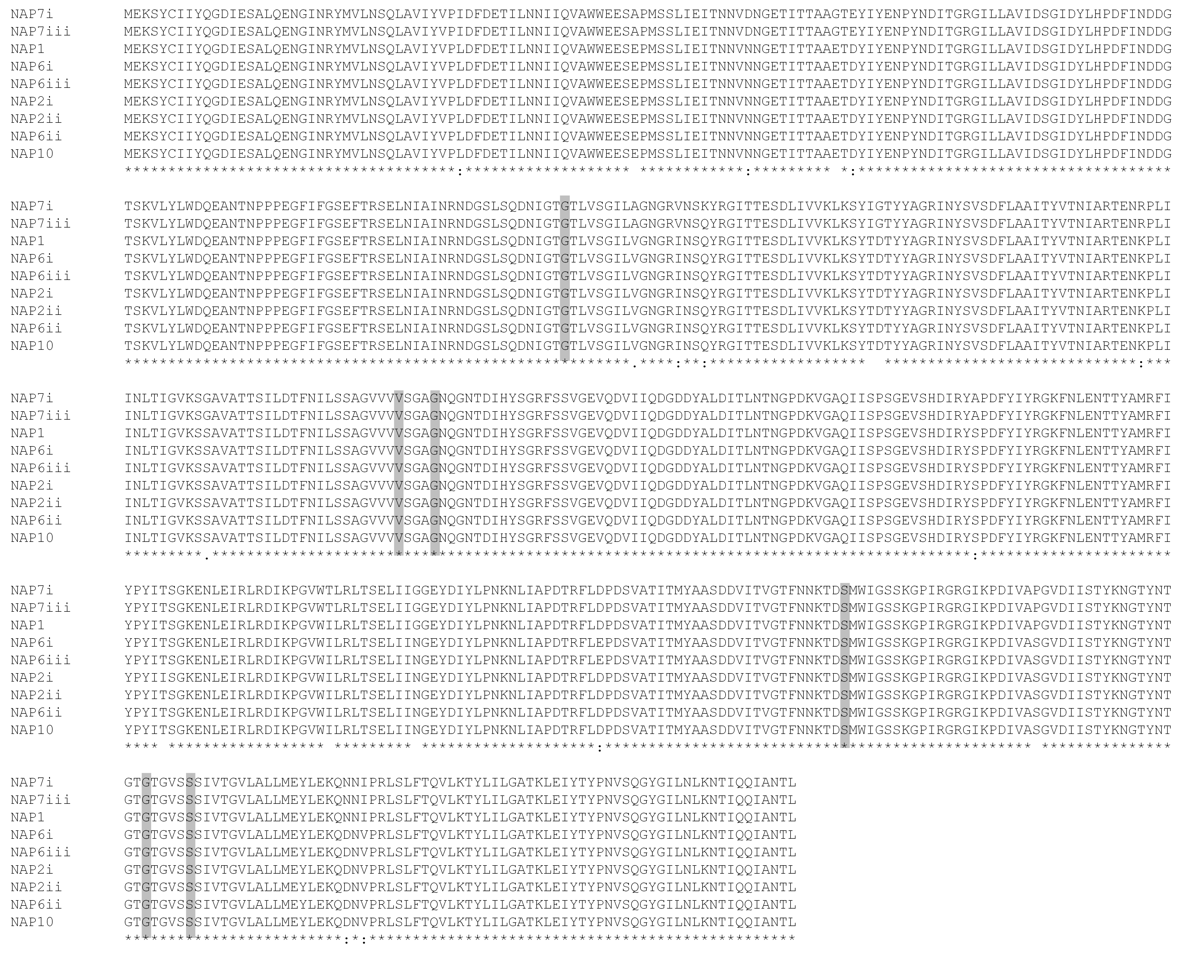

Supplement: S4 Fig — Protein sequences from 9 isolates. Roman numerals represent isolate number within PFGE type. Highlighted loci have previously been reported to be essential for germination [37]. Legend: * = conserved residue;: = conservative mutation;. = semi-conservative mutation; blank = non-conservative mutation; and NAP = North American pulsed-field gel electrophoresis (PFGE) type. (TIF) [file pone.0147210.s004.tif]
