# Supplementary material for: Changes in Colonic Bile Acid Composition following Fecal Microbiota Transplantation Are Sufficient to Control Clostridium difficile Germination and Growth
Source: PLoS One. 2016 Jan 20;11(1):e0147210. doi: 10.1371/journal.pone.0147210 (PMC4720481; doi:10.1371/journal.pone.0147210)
Supplement: S1 Table — (DOCX) [file pone.0147210.s005.docx]

**S1 Table. *C. difficile* *cspC* PCR primers used in this study.**

| **Primer** | **Position on *cspC* gene (5’ end)** | **Sequence**  **(5’ to 3’)** |
| --- | --- | --- |
| Forward primer 1A | 4 | AACACGTATGCAACTATAACTG |
| Forward primer 1B | 650 | CAAATCCACCTCCAGAAGGT |
| Forward primer 1C | 1300 | GAAAATACTACTTATGCAATGAG |
| Reverse primer 1 | 2146 | AAAATTCATAAGTCTAAATTATAATG |
| Forward primer 2A | 22 | ACTGGTACAGCAGCAGCA |
| Forward primer 2B | 628 | TTATGGGACCAAGAAGCAA |
| Forward primer 2C | 1250 | ATATAAGGTATTCTCCAGAC |
| Reverse primer 2 | 2167 | ACTTTTAGCAATTTTACAAATAAA |
