# Supplementary material for: Changes in Colonic Bile Acid Composition following Fecal Microbiota Transplantation Are Sufficient to Control Clostridium difficile Germination and Growth
Source: PLoS One. 2016 Jan 20;11(1):e0147210. doi: 10.1371/journal.pone.0147210 (PMC4720481; doi:10.1371/journal.pone.0147210)
Supplement: S2 Table — (DOCX) [file pone.0147210.s006.docx]

**S2 Table. Mean relative OD_600_ of spores from 10 isolates after 20 min exposure to bile acids**.

|  | **Bile Acid (mM)** | **PFGE type (isolate no.)^a^** | | | | | | | | | |
| --- | --- | --- | --- | --- | --- | --- | --- | --- | --- | --- | --- |
| **Bile acid** |  | **NAP1** | **NAP2 (i)** | **NAP2 (ii)** | **NAP6 (i)** | **NAP6 (ii)** | **NAP6 (iii)** | **NAP7 (i)** | **NAP7 (ii)** | **NAP7 (iii)** | **NAP10** |
| **Pre-FMT Bile Acids** |  | 0.90 | 0.93 | 0.88 | 0.90 | 0.88 | 0.86 | 0.60 | 0.65 | 0.62 | 0.88 |
| SEM ^h^ |  | *0.02* | *0.08* | *0.02* | *0.03* | *0.01* | *0.01* | *0.01* | *0.01* | *0.02* | *0.01* |
| **Post-FMT Bile Acids** |  | 1.01 | 1.00 | 1.02 | 1.09 | 1.07 | 1.02 | 0.96 | 1.04 | 1.05 | 1.11 |
| SEM |  | *0.02* | *0.06* | *0.03* | *0.03* | *0.02* | *0.01* | *0.02* | *0.03* | *0.01* | *0.03* |
| **BHIS^b^** |  | 0.97 | 1.03 | 1.10 | 1.01 | 1.10 | 1.07 | 1.10 | 0.97 | 1.08 | 0.94 |
| SEM |  | *0.03* | *0.03* | *0.05* | *0.06* | *0.07* | *0.04* | *0.07* | *0.02* | *0.05* | *0.06* |
| **TA^c^** | 0.5 | 0.66 | 0.73 | 0.76 | 0.90 | 0.84 | 0.89 | 0.70 | 0.69 | 0.62 | 0.67 |
| SEM |  | *0.02* | *0.02* | *0.03* | *0.02* | *0.03* | *0.05* | *0.01* | *0.02* | *0.00* | *0.03* |
|  | 2 | 0.52 | 0.47 | 0.48 | 0.62 | 0.69 | 0.60 | 0.56 | 0.43 | 0.43 | 0.55 |
| SEM |  | *0.01* | *0.03* | *0.01* | *0.02* | *0.01* | *0.01* | *0.02* | *0.03* | *0.01* | *0.01* |
| **CA^d^** | 0.5 | 0.98 | 1.05 | 1.02 | 0.94 | 0.94 | 1.03 | 0.73 | 0.83 | 0.90 | 1.00 |
| SEM |  | *0.01* | *0.08* | *0.04* | *0.01* | *0.03* | *0.03* | *0.10* | *0.04* | *0.06* | *0.04* |
|  | 1 | 0.89 | 1.01 | 0.97 | 1.03 | 0.94 | 0.93 | 0.7 | 0.66 | 0.79 | 0.90 |
| SEM |  | *0.03* | *0.05* | *0.03* | *0.03* | *0.03* | *0.04* | *0.00* | *0.05* | *0.02* | *0.04* |
|  | 2 | 0.94 | 0.96 | 0.93 | 0.93 | 0.91 | 0.92 | 0.60 | 0.71 | 0.71 | 0.95 |
| SEM |  | *0.02* | *0.04* | *0.01* | *0.04* | *0.04* | *0.03* | *0.01* | *0.04* | *0.02* | *0.04* |
| **CDCA^e^** | 0.25 | 1.02 | 1.00 | 1.01 | 0.93 | 1.03 | 0.93 | 0.99 | 0.92 | 0.97 | 0.99 |
| SEM |  | *0.06* | *0.06* | *0.06* | *0.09* | *0.04* | *0.02* | *0.03* | *0.02* | *0.03* | *0.03* |
|  | 0.5 | 1.00 | 1.08 | 1.00 | 0.95 | 0.94 | 0.97 | 1.09 | 0.98 | 0.91 | 1.05 |
| SEM |  | *0.01* | *0.07* | *0.02* | *0.03* | *0.02* | *0.03* | *0.01* | *0.02* | *0.05* | *0.07* |
|  | 1 | 1.01 | 1.04 | 1.09 | 1.06 | 0.96 | 1.03 | 1.11 | 1.13 | 1.00 | 1.06 |
| SEM |  | *0.02* | *0.03* | *0.01* | *0.04* | *0.03* | *0.01* | *0.04* | *0.04* | *0.07* | *0.07* |
|  | 2 | 0.91 | 0.90 | 1.07 | 0.98 | 0.96 | 0.98 | 0.83 | 0.97 | 0.94 | 0.77 |
| SEM |  | *0.03* | *0.02* | *0.05* | *0.01* | *0.01* | *0.05* | *0.04* | *0.04* | *0.01* | *0.01* |
| **CDCA+2 mM TA** | 0.25 | 0.57 | 0.61 | 0.56 | 0.64 | 0.64 | 0.70 | 0.57 | 0.59 | 0.62 | 0.57 |
| SEM |  | *0.01* | *0.04* | *0.02* | *0.01* | *0.01* | *0.02* | *0.01* | *0.01* | *0.01* | *0.01* |
|  | 0.5 | 0.84 | 0.71 | 0.86 | 0.79 | 0.74 | 0.73 | 0.76 | 0.67 | 0.72 | 1.11 |
| SEM |  | *0.07* | *0.05* | *0.03* | *0.07* | *0.01* | *0.01* | *0.01* | *0.03* | *0.02* | *0.11* |
|  | 1 | 1.05 | 0.86 | 1.02 | 1.01 | 0.85 | 0.91 | 0.87 | 0.87 | 0.83 | 1.10 |
| SEM |  | *0.02* | *0.10* | *0.12* | *0.01* | *0.01* | *0.03* | *0.00* | *0.02* | *0.04* | *0.04* |
|  | 2 | 1.08 | 0.87 | 1.03 | 1.10 | 0.96 | 1.04 | 0.85 | 0.95 | 0.87 | 0.96 |
| SEM |  | *0.04* | *0.06* | *0.04* | *0.01* | *0.04* | *0.01* | *0.03* | *0.03* | *0.01* | *0.05* |
| **DCA^f^** | 0.5 | 1.04 | 1.06 | 1.02 | 0.93 | 0.89 | 0.93 | 0.72 | 0.77 | 0.92 | 1.03 |
| SEM |  | *0.01* | *0.02* | *0.02* | *0.01* | *0.01* | *0.04* | *0.01* | *0.01* | *0.01* | *0.05* |
|  | 1 | 1.00 | 1.05 | 0.99 | 1.03 | 0.90 | 0.95 | 0.78 | 0.76 | 0.89 | 1.11 |
| SEM |  | *0.01* | *0.04* | *0.05* | *0.02* | *0.05* | *0.04* | *0.08* | *0.07* | *0.03* | *0.06* |
|  | 2 | 1.12 | 1.00 | 0.83 | 0.97 | 0.86 | 0.80 | 0.98 | 0.71 | 0.85 | 1.05 |
| SEM |  | *0.07* | *0.01* | *0.05* | *0.01* | *0.02* | *0.04* | *0.02* | *0.03* | *0.05* | *0.03* |
| **LCA^g^** | 0.5 | 1.08 | 1.01 | 1.05 | 1.04 | 0.99 | 1.05 | 0.97 | 1.00 | 1.00 | 0.98 |
| SEM |  | *0.02* | *0.01* | *0.02* | *0.03* | *0.02* | *0.04* | *0.03* | *0.02* | *0.02* | *0.04* |
|  | 1 | 1.05 | 1.06 | 1.03 | 1.02 | 1.00 | 1.00 | 1.04 | 1.03 | 0.94 | 0.96 |
| SEM |  | *0.04* | *0.07* | *0.02* | *0.02* | *0.03* | *0.02* | *0.03* | *0.00* | *0.04* | *0.01* |
|  | 2 | 1.05 | 1.03 | 0.88 | 1.00 | 1.03 | 1.10 | 0.96 | 1.01 | 0.94 | 1.02 |
| SEM |  | *0.06* | *0.11* | *0.11* | *0.04* | *0.03* | *0.05* | *0.04* | *0.02* | *0.03* | *0.03* |

^a^PFGE = Pulsed-field gel electrophoresis, ^b^BHIS = BHI broth with yeast extract and 0.1% L-cysteine, ^c^TA = taurocholate, ^d^CA = cholate, ^e^CDCA = chenodeoxycholic acid, ^f^DCA = deoxycholate, ^g^LCA = lithocholic acid.

^h^Bottom row of each group displays the standard error of the mean (SEM) for the mean listed in the cell above.
